# Supplementary material for: Neuroticism Overestimated? Neuroticism Versus Hypertonia, Pain and Rehabilitation Outcomes in Post-Spinal Cord Injury Patients Rehabilitated Conventionally and with Robotic-Assisted Gait Training
Source: Brain Sci. 2024 Nov 18;14(11):1153. doi: 10.3390/brainsci14111153 (PMC11592360; doi:10.3390/brainsci14111153)
Supplement: Supplementary file 1 [file brainsci-14-01153-s001.zip › brainsci-3300596-supplementary.pdf]

Supplementary Materials

In order to exclude a factor related to participants' level of motivation and engagement, The Hopkins Rehabilitation Engagement Rating Scale—Reablement Version (HRERS-RV) was used to measure. The scale has good validity and reliability. (However, the analyses so far show that there is a so-called ceiling effect in the measurement. This may be due to imprecise measurement of the dependent variables or to variables that interfere with internal validity). HRERS-RV was scored by medical or paramedical staff at the end of the 7-week rehabilitation programme. One hundred and ten people participated in the study. From this group, individuals with high and low levels of neuroticism traits were selected. This was done on the basis of the NEO-PI-R Inventory scores (converted to sten scores (i.e. 1–4 sten—low (no) neuroticism, 7–10—high neuroticism). On this basis, 78 individuals were included in the study, 57 with low (no) neuroticism and 21 with high neuroticism.

Table S1. Mean scores obtained on the HRERS-RV scale by subjects with high and low levels of neuroticism.

	**Low Neuroticism**		**High Neuroticism**	
**HRERS-RV Score**	***M***	**%**	***M***	**%**
	21.79	87.16%	22.05	88.19%
